# Tendon-muscle-nerve disconnection in chronic large and massive posterosuperior rotator cuff tears: a targeted narrative mechanistic review and hypothesis-generating framework

**DOI:** 10.3389/fcell.2026.1928020

**Published:** 2026-09-14

**Authors:** Tao Chen, Jinghui Song, Liang Ma

**Affiliations:** Orthopedics, Jingzhou Hospital Affiliated to Yangtze University, Jingzhou, China

**Keywords:** fatty infiltration, fibro-adipogenic progenitors, muscle atrophy, quantitative MRI, rotator cuff tear, suprascapular nerve, translational research

## Abstract

Anatomical repair of chronic large or massive posterosuperior rotator cuff tears does not invariably restore strength, endurance, or patient-reported function. This targeted narrative mechanistic review synthesizes cuff-specific human studies, rotator cuff animal models, and explicitly labelled non-cuff skeletal-muscle evidence to examine persistent dysfunction after repair. Its primary purpose is to calibrate evidence across mechanical load-transfer failure, selected neural modifiers, and stromal remodeling; its secondary purpose is to propose a falsifiable, hypothesis-generating tendon-muscle-nerve (TMN) framework. Human evidence is strongest for the prognostic association of fatty infiltration, atrophy, muscle volume, and tendon integrity. Suprascapular neuropathy is documented in selected patients but is not present in most chronic massive tears. Cuff-specific evidence for neuromuscular-junction remodeling is sparse and conflicting, whereas fibro-adipogenic progenitor, macrophage, extracellular-vesicle, and metabolic-fate mechanisms remain predominantly animal, *ex-vivo*, or extrapolative. We therefore propose that mechanical burden, defined neural modifiers, and candidate biological stage be measured separately and initially retained as continuous variables. Proposed categories and quantitative anchors require prospective testing for reliability, longitudinal stability, incremental prognostic value, and treatment-effect heterogeneity. The TMN construct is not a validated diagnosis, staging system, or treatment algorithm. Mechanical restoration remains the clinical foundation; neural and stromal interventions remain selective or preclinical research hypotheses.

## Highlights

- Dominant mechanism and biological stage should be recorded as separate dimensions.
- Peripheral neuropathy, pain-related central inhibition, and motor-unit recruitment are distinct measurable domains; NMJ remodeling remains a hypothesis-level research question.
- Human evidence is strongest for muscle-quality prognosis; most NMJ, extracellular-vesicle and FAP-redirection mechanisms remain preclinical or extrapolative.
- FAP and ECM remodeling may represent one candidate stromal link between altered loading, immune signaling, and fibrofatty replacement, but cuff-specific human evidence remains limited.
- The framework requires prospective validation beyond tear size, retraction, Goutallier grade and tendon healing.

## 1 Introduction and conceptual framework

The clinical paradox motivating this review is straightforward: tendon healing matters, yet it does not guarantee biological recovery. In chronic large and massive tears, even a healed repair may coexist with persistent weakness, limited endurance, pain-related inhibition, and disappointing function. The more difficult question is not simply whether the tendon can be reattached, but whether the remaining muscle is still contractile, recruitable, metabolically responsive, and capable of transmitting force.

Fatty infiltration, atrophy, and fibrosis provide the clearest clinical clue. The Goutallier classification made fatty degeneration a reproducible imaging feature, and subsequent MRI studies associated fatty degeneration, atrophy, and tear chronicity with reparability, healing, and outcome (Goutallier et al., 1994; Fuchs et al., 1999; Goutallier et al., 2003; Patte, 1990; Thomazea et al., 1997; Zanetti et al., 1998; Nakagaki et al., 1995). Notably, these muscle abnormalities often persist after repair and accompany poorer clinical results and lower force (Gladstone et al., 2007; Gerber et al., 2007). Studies of large and massive tears reach a similar conclusion from another direction: tendon integrity is necessary, but it does not account for all variation in postoperative function (Galatz et al., 2004; Harryman et al., 1991; Gerber et al., 2000).

Here, TMN disconnection is used solely as an organizing research construct for separately measurable failures of tendon load transfer, muscle contractility, neural activation, and stromal homeostasis. It does not denote a uniform lesion, a validated staging system, or a predetermined causal sequence, and it is not intended as a diagnostic label or treatment algorithm.

Our primary focus is chronic large and massive posterosuperior tears involving the supraspinatus and infraspinatus. Definitions of a massive tear differ across studies, variously relying on a diameter greater than 5 cm, involvement of at least two tendons, or topographic criteria (Lädermann et al., 2016). We therefore retain the definition used in each source and use large/massive as a scope descriptor, not as a new threshold. Acute traumatic tears, isolated subscapularis or teres minor disease, postoperative failure, and cuff-tear arthropathy may share elements of this model, but their trajectories should not be assumed to be identical.

The purpose of this targeted narrative review is to synthesize what is established in human cuff disease, identify where cuff-specific animal experiments provide model-limited perturbational evidence, and distinguish these findings from non-cuff skeletal-muscle extrapolation. The proposed framework is intended to generate testable hypotheses and minimum datasets rather than to establish a new diagnosis.

We therefore organize the synthesis around two independent research questions. First, is the observed pattern most consistent with mechanical unloading, a measured neural modifier, or both? Second, do imaging and function suggest candidate early stress, intermediate remodeling, or advanced fibrofatty replacement? These labels are probabilistic research descriptors and require prospective validation.

The subsequent sections proceed from the strongest evidence for mechanical load-transfer failure and human muscle-quality prognosis to selected neural findings, candidate stromal mechanisms, unvalidated temporal descriptors, research phenotyping, and translational boundaries.

Established structural variables remain the starting point for both clinical and experimental work: tear size, tendon involvement, retraction, topography, muscle volume, and fatty infiltration (Patte, 1990; Lädermann et al., 2016). The TMN approach asks a narrower, testable question: do separately measured mechanical, neural, and stromal variables add reproducible prognostic information, and does biological stage modify the response to structural repair?

Fatty infiltration is not merely a radiological descriptor. It signals loss of muscle quality and may contribute directly to poor function. CT and MRI grading systems provided the historical foundation; quantitative MRI, CT density, three-dimensional muscle volume, and regional fat mapping now offer a more detailed view of spatial heterogeneity (Goutallier et al., 1994; Goutallier et al., 2003; Zanetti et al., 1998; Kuzel et al., 2013; Valencia et al., 2018). This distinction matters because mechanical unloading and neurogenic injury may not produce the same distribution of fat.

Natural-history studies show that untreated or enlarging tears may progress both in size and in muscle degeneration. Fatty infiltration and atrophy are not explained by age alone; they also track with chronicity, tendon involvement, retraction, and symptomatic progression (Melis et al., 2010; Mall et al., 2010; Keener et al., 2015; Safran et al., 2011; Yamaguchi et al., 2001). The muscle therefore has a trajectory of its own. A tendon lesion establishes the pathologic setting, but the muscle and interstitium continue to remodel even as symptoms fluctuate.

Clinical outcome studies connect fatty infiltration with function. Fatty degeneration predicts inferior strength and repair integrity, histological studies show myofiber degeneration, fibrosis, and cellular changes in advanced human disease, and biomechanical work links higher fat fraction with reduced force-generating capacity (Gladstone et al., 2007; Gerber et al., 2007; Galatz et al., 2004; Bogd et al., 2021; Bowen et al., 2025; Santago et al., 2016; Gibbons et al., 2017; Valencia et al., 2018). A successful repair consequently has two relevant endpoints: structural tendon continuity and reconnection of a muscle that can generate and transmit force.

Figure 1 provides a conceptual overview; the subsequent sections evaluate each component according to evidence source and strength.

**FIGURE 1:**
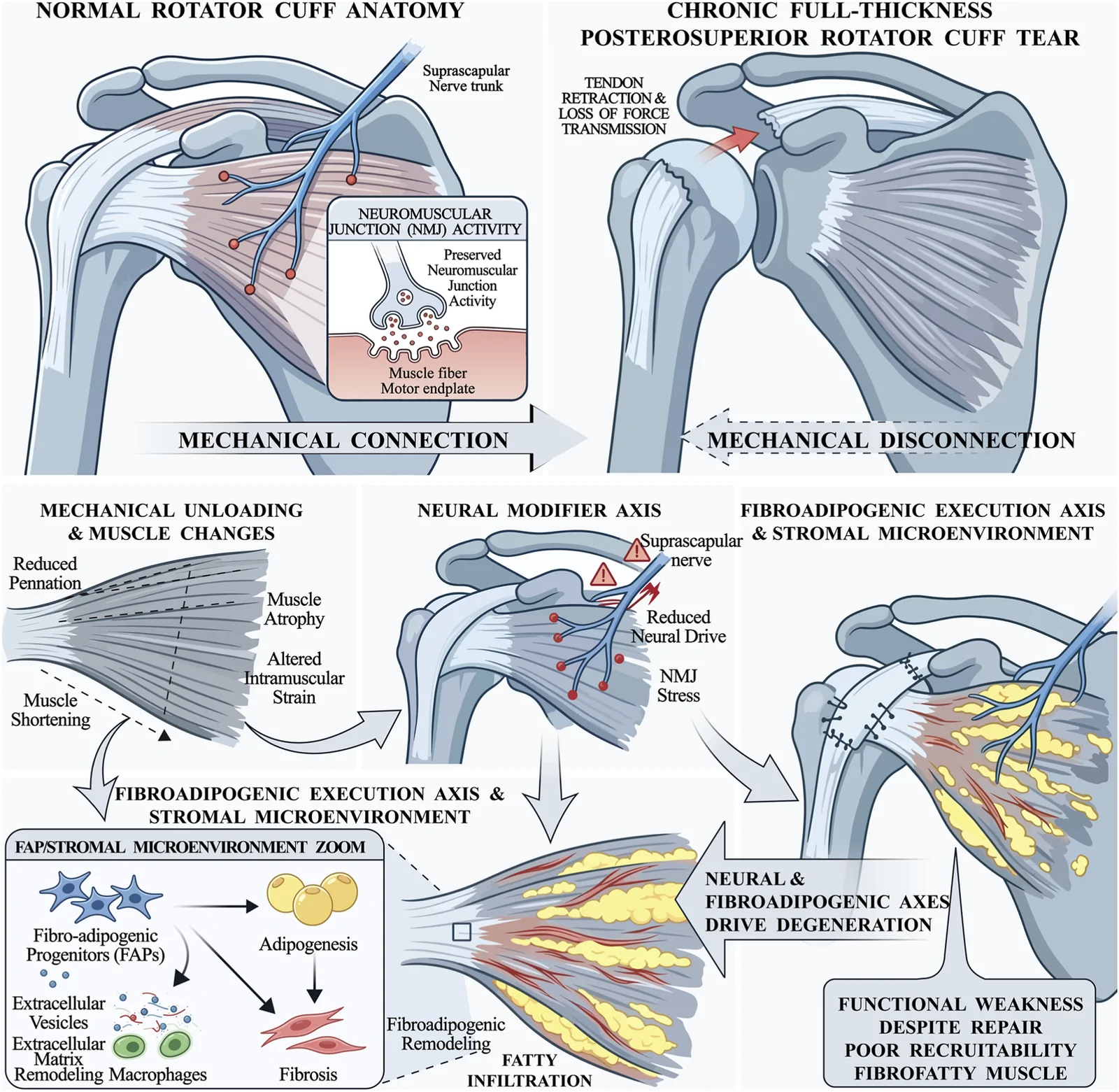
Hypothesis-generating conceptual map of tendon-muscle-nerve dysfunction in chronic large or massive posterosuperior rotator cuff tears. Mechanical load-transfer failure has the strongest cuff-specific human and animal support. Documented peripheral neural abnormalities may modify selected cases; NMJ remodeling and FAP/immune-metabolic links are predominantly preclinical or extrapolative. Arrows denote proposed relationships and do not imply equal evidentiary strength or a deterministic sequence.

## 2 Mechanical disconnection: force-transmission failure and muscle remodeling

Loss of force transmission is the most immediate consequence of a chronic full-thickness tear. In an intact cuff, force generated by the supraspinatus and infraspinatus is transmitted through the intramuscular connective tissue and myotendinous unit to the tendon insertion on the greater tuberosity of the humerus. Rupture interrupts that path. Muscle fibers may continue to contract, but their force is no longer conveyed efficiently to bone. This is more specific than simple disuse: the route by which the muscle performs mechanical work has failed.

The biomechanical literature supports this interpretation. The rotator cable and crescent concept explains how load may be redistributed across the cuff, whereas partial-repair strategies seek to restore force couples when complete anatomical coverage is not possible (Burkhart et al., 1993; Burkhart et al., 2003; Burkhart, 1997). The biological corollary is plausible: when force cannot be transmitted, the muscle loses tensile feedback, sarcomere-length homeostasis, and activity-dependent metabolic demand. Thus, even a moving shoulder may contain a chronically under-loaded muscle.

Animal studies provide causal support for this mechanical pathway. Tendon release or tenotomy can produce muscle atrophy, fatty infiltration, and fibrosis without deliberate nerve injury. Across sheep, rat, and mouse models, tendon release, delayed repair, and creation of large tears lead to persistent changes in muscle architecture and composition (Gerber et al., 2004; Meyer et al., 2004; Luan et al., 2015; Liu et al., 2011; Liu et al., 2012; Kim et al., 2012; Gumucio et al., 2014; Wang et al., 2018a; Rowshan et al., 2010). Differences in quadruped anatomy, loading, and chronicity limit direct translation, but the repeated result is important: tendon injury alone can initiate muscle degeneration.

The disruption is also architectural. Shortening, loss of pennation, altered passive tension, and fibrotic stiffening may leave a muscle that can be mobilized surgically but remains physiologically compromised. Once this remodeling is established, restoring tendon length may recover anatomy without normalizing intramuscular strain. This helps explain why early restoration of tension may not be biologically equivalent to late repair.

## 3 Neural dysfunction: established peripheral findings and candidate modifiers

### 3.1 Peripheral suprascapular neuropathy

The suprascapular nerve warrants attention because it supplies both the supraspinatus and infraspinatus. Medial tendon retraction may increase traction at the suprascapular or spinoglenoid notch. Nevertheless, anatomical plausibility alone is not evidence of electrophysiologically confirmed neuropathy.

Direct human evidence is available, although chiefly in selected subgroups. Suprascapular neuropathy has been documented in some massive tears, and recovery after repair or decompression has been reported (Boykin et al., 2010; Mallon et al., 2006; Costouros et al., 2007; Lafosse et al., 2007). By contrast, prospective EMG studies have not found neuropathy in most massive tears, and comparative data do not support routine decompression (Collin et al., 2014; Gerber et al., 2020). Peripheral neuropathy therefore needs to be demonstrated, not inferred from tear size or fatty infiltration.

Imaging and experimental studies help define the limits of this association. Chronic cuff tears and suprascapular neuropathy may produce different patterns of atrophy and fat (Beeler et al., 2013); severe neurogenic fatty infiltration can occur despite intact tendons (Leclere et al., 2014); and isolated tendon or nerve injury can independently induce fatty infiltration, atrophy, and fibrosis in animals (Mistretta et al., 2025; Eshima et al., 2023). Taken together, these observations support a neural modifier in a subset of cases, not a universal neural cause.

When present, a peripheral nerve abnormality may intensify degeneration, broaden its distribution, or limit recovery. Compression, traction injury, and denervation need to be examined as component-specific questions. They should not be conflated with pain inhibition, reduced voluntary drive, or remodeling at the neuromuscular junction.

### 3.2 Pain-related central inhibition and motor-unit recruitment

A patient may also fail to activate the cuff fully in the absence of a peripheral nerve lesion. Pain-related central inhibition and altered motor control can reduce voluntary drive, whereas motor-unit recruitment is a separate physiological level that may be assessed through interference-pattern analysis, motor-unit number estimation, or activation testing. Both are plausible explanations for weakness that appears disproportionate to structure, but direct longitudinal evidence in chronic large/massive posterosuperior tears remains sparse.

EMG/NCS is useful for confirming selected peripheral abnormalities, but its interpretive limits matter. A normal result does not exclude mechanical unloading or pain-related inhibition, and an abnormal result does not establish failure of the neuromuscular junction. Future studies need to report these neural levels separately and relate each to objective strength, endurance, pain, and the regional imaging pattern (Boykin et al., 2010; Mallon et al., 2006; Costouros et al., 2007; Lafosse et al., 2007; Collin et al., 2014; Gerber et al., 2020; Beeler et al., 2013; Leclere et al., 2014; Mistretta et al., 2025; Eshima et al., 2023).

### 3.3 Hypothesis-generating NMJ remodeling: general skeletal-muscle extrapolation

Cuff-specific evidence for NMJ remodeling remains sparse and inconsistent, and comparable longitudinal data in human chronic cuff tears are unavailable. Most mechanistic information on motor-endplate remodeling, acetylcholine-receptor organization, and excitation-contraction coupling derives from aging, denervation, or neuromuscular disease outside the rotator cuff (Tintignac et al., 2015; Li et al., 2018; R et al., 2014; Blaauw et al., 2013). NMJ morphology should therefore be treated as a hypothesis-generating research endpoint—not an established driver, a phenotyping criterion, or an inference from conventional EMG/NCS.

### 3.4 Neural-mechanical convergence

Mechanical unloading and reduced activation may ultimately create a similar low-tension, low-demand microenvironment. Studies in general skeletal muscle connect reduced loading and altered growth signaling with the autophagy-lysosome and ubiquitin-proteasome systems, as well as Akt/mTOR and FoxO pathways (Sandri, 2013; Bonaldo and Sandri, 2013; Egerman and Glass, 2014; Schiaffino et al., 2013; Bodine et al., 2001; Sandri et al., 2004; Jorgenson et al., 2020). These findings establish biological plausibility, but they do not show that one convergence pathway accounts for human cuff disease.

One animal study illustrates the point. In a combined massive-tear and suprascapular-nerve-injury model, mTOR-SREBP-1-PPAR-gamma signaling was associated with adipogenesis, and mTOR inhibition reduced fatty infiltration (Joshi et al., 2013). The study identifies a modifiable pathway in that experimental setting; it does not establish a master switch or a validated target in patients.

Tendon-only and nerve-only injuries can each generate fat, while their combination may produce more extensive or differently distributed change. Whether such patterns form reproducible human clusters remains unresolved. Prospective studies will need to account for age, metabolic status, pain, and tear morphology before assigning the difference to neural-mechanical interaction.

Potential early signals include loss of muscle volume, edema-like or altered T2 signal, heterogeneous fat fraction, reduced activation, and weakness out of proportion to the structural lesion (Zbinden et al., 2026). None is specific. Their value is more likely to emerge from a multimodal panel than from use as isolated diagnostic criteria.

### 3.5 Neural-directed strategies: compartment, timing, and combination

Lesion-directed treatment is biologically most plausible when a localizable and potentially reversible neuropathy has been demonstrated. Selected human studies do not support routine suprascapular nerve decompression during cuff repair in the absence of a documented lesion (Collin et al., 2014; Gerber et al., 2020). A mouse compression-and-release study suggests that timely relief can improve reinnervation and aspects of muscle remodeling, but that model did not include a tendon tear or repair (Wang et al., 2018b). Neural management should therefore be lesion-specific rather than prophylactic.

Activity-maintenance strategies—including graded activation and neuromuscular electrical stimulation—could be evaluated as ways to limit disuse or under-recruitment while protecting the repair. They should not be described as synaptic rescue, because postoperative human evidence has not established restoration of cuff NMJ biology or durable strength recovery. Timing and intensity must be tested against tendon-healing constraints.

At a molecular level, preservation of agrin–LRP4–MuSK signaling and acetylcholine-receptor clustering derives mainly from non-cuff NMJ biology (Tintignac et al., 2015; Li et al., 2018; R et al., 2014; Blaauw et al., 2013), whereas denervation-associated IL-6–STAT3 signaling links neural loss to stromal and fibrotic responses in general skeletal muscle (Madaro et al., 2018). These are hypothesis-generating platforms, not cuff-specific therapies. Future experiments should measure proximal neural outcomes together with inflammation, FAP state, ECM remodeling, tendon healing, and function. Neural monotherapy is most plausible for an early documented lesion; mixed phenotypes would generally require mechanical restoration, with neural or activity-directed treatment evaluated as an adjunct.

## 4 Stromal and extracellular-matrix remodeling: human observations and candidate cellular mechanisms

Human cuff tissue and imaging studies document myofiber loss, fat accumulation, atrophy, and fibrosis, but they do not identify one cellular pathway as the dominant cause of chronic degeneration (Gladstone et al., 2007; Gerber et al., 2007; Galatz et al., 2004; Bogd et al., 2021; Bowen et al., 2025; Santago et al., 2016; Gibbons et al., 2017; Valencia et al., 2018). Fibro-adipogenic progenitors (FAPs) constitute one plausible candidate stromal axis. Their regenerative, adipogenic, and fibrogenic potential is established principally in non-cuff skeletal muscle, whereas cuff-specific support derives largely from animal lineage and perturbation studies (Joe et al., 2010; Uezumi et al., 2010; Uezumi et al., 2011; Mozzetta et al., 2013; Heredia et al., 2013; Lemos et al., 2015; Madaro et al., 2018; Wosczyna et al., 2019; Biferali et al., 2019; Molina et al., 2021; Contreras et al., 2021; Negroni et al., 2022; Liu et al., 2016; Shirasawa et al., 2017; Agha et al., 2021; Lee et al., 2020a; Davies et al., 2022; Lin et al., 2023; Zhang et al., 2025; Flores-Opazo et al., 2024). Longitudinal human data linking FAP state to prognosis or reversibility are lacking.

Foundational skeletal muscle studies demonstrated that PDGFR-alpha-positive interstitial progenitors are distinct from satellite cells and can form ectopic fat, that fibrosis and adipogenesis can arise from related mesenchymal progenitors, and that FAP survival or clearance depends on immune and cytokine cues (Joe et al., 2010; Uezumi et al., 2010; Uezumi et al., 2011; Mozzetta et al., 2013; Heredia et al., 2013; Lemos et al., 2015; Madaro et al., 2018; Wosczyna et al., 2019). These data provide the conceptual basis for interpreting rotator cuff fatty infiltration as a regulated cell-fate process rather than passive lipid deposition.

Cuff-specific animal studies illustrate both biological plausibility and translational distance. In a severe combined mouse model involving suprascapular denervation, full cuff tendon transection, and humeral-head resection, oral imatinib at 30 mg/kg 5 days per week suppressed early adipogenic gene expression, PDGFR-alpha-positive cells, and histological fat by two to 4 weeks (Shirasawa et al., 2017). The model and early treatment timing test prevention of fat development, not reversal of established human disease, and imatinib is not FAP-specific. Comparisons of cuff- and limb-derived murine FAPs further suggest tissue-specific adipogenic behavior, but do not establish intrinsic susceptibility in human cuff muscle (Agha et al., 2021).

Cell number alone may be less informative than cell fate. Some FAP states support myogenesis; others form white adipocytes, deposit collagen-rich matrix, or enter beige/brown-like adipogenic programs with greater metabolic activity. Tear size, chronic unloading, and inflammation may shift this balance (Agha et al., 2021; Lee et al., 2020a; Davies et al., 2022; Lin et al., 2023; Zhang et al., 2025; Flores-Opazo et al., 2024). Such fate bias is likely to be more biologically meaningful than a simple positive-or-negative FAP marker.

In general skeletal-muscle injury, macrophage states influence debris clearance, satellite-cell activity, and FAP survival or differentiation (Arno et al., 2007; Tidball, 2017; Chazaud, 2014; Wynn and Vannella, 2016). Whether the same temporal sequence governs chronic human cuff degeneration is unknown, and the M1/M2 binary does not capture the full diversity or timing of macrophage states.

Cuff-specific evidence remains preclinical. In a mouse supraspinatus/infraspinatus tendon-transection-plus-denervation model, 2 × 10^9 M0-, M1-, or M2-macrophage-derived exosomes were injected locally at injury and the supraspinatus was assessed 6 weeks later. In this small experiment, M2-derived exosomes were associated with less fatty infiltration, fibrosis, and atrophy and with greater UCP1-related beige differentiation (Liu et al., 2023). The prophylactic, single-model result identifies a candidate signaling route; it does not establish extracellular-vesicle therapy, a universally beneficial macrophage state, or reversal of established disease.

The familiar M1/M2 binary also understates the dynamic and multidimensional nature of macrophage states. Age, metabolic health, mechanical load, and damage signals may all modify macrophage-FAP interactions (Arno et al., 2007; Tidball, 2017; Chazaud, 2014; Wynn and Vannella, 2016; Liu et al., 2023; Greenberg and Reeves, 2021). Future studies will need to combine state-resolved markers with functional assays rather than infer benefit from a single polarization label.

Metabolism may connect these immune signals to the eventual fat phenotype. Beige adipogenesis, UCP1 expression, beta-adrenergic signaling, and mitochondrial activity could indicate a more plastic adipose state than mature white fat. Conversely, low metabolic demand, insulin resistance, aging, and persistent inflammation may favor white adipogenesis and fibrosis. Measuring only a reduction in visible fat would miss this functional heterogeneity.

Fibrosis deserves attention alongside fatty infiltration. Collagen accumulation can alter passive stiffness, intramuscular force transmission, vascular diffusion, satellite-cell behavior, and surgical mobilization. A cuff muscle replaced by both fat and collagen is not simply weak; its mechanical and biological connection to the repair construct has been compromised.

Skeletal-muscle fibrosis reflects dysregulated repair, including TGF-beta-associated signaling, persistent fibroblast/FAP activation, and abnormal extracellular-matrix turnover (Gillies and Lieber, 2011; Lieber and Ward, 2013; Mann et al., 2011; Serrano and Muñoz-Cánoves, 2010; Bentzinger et al., 2012). In chronic large/massive posterosuperior tears, fibrosis may limit restoration of tendon length, increase repair tension, and reduce postoperative adaptability. It may also complicate imaging-based interpretation: two muscles with similar fat fractions may perform differently if one contains substantially more collagen.

The relevant tissue endpoint is therefore fibroadipogenic remodeling: the simultaneous loss of contractile tissue and its replacement by adipogenic and collagen-rich interstitium. Whether cellular state predicts reversibility better than quantitative imaging is an important question for future studies, not an established clinical claim.

## 5 Candidate biological stages and limits of reversibility

Biological stage and dominant mechanism are conceptually separate, but the proposed stages remain unvalidated research descriptors. Calendar time alone is insufficient because tear morphology, age, metabolic health, pain, neural involvement, and loading history may alter the rate and pattern of remodeling.

A candidate early-stress phenotype would be expected to show reduced tension or activation with limited tissue replacement, whereas a candidate intermediate-remodeling phenotype would show measurable regional fat or collagen accumulation with residual contractile reserve. FAP or neural changes may accompany either pattern but should not be inferred without direct measurement. No validated human threshold currently separates these states.

Several stromal, immune, and metabolic pathways are modifiable in preclinical systems (Liu et al., 2016; Agha et al., 2021; Lin et al., 2023; Liu et al., 2023; Shirasawa et al., 2021; Lee et al., 2020b; Lee et al., 2020c; Wang et al., 2021; Wang et al., 2020; Iio et al., 2023; Zhou et al., 2023; Zhou et al., 2024; Liu et al., 2022; Stamiris et al., 2024; Rodeo, 2007). Because most treatments were initiated at or near acute injury, those experiments correspond more closely to prevention or early remodeling than to reversal of established chronic degeneration.

At the proposed advanced end of the spectrum, high fat burden, collagen-rich fibrosis, severe atrophy, and low contractile reserve are expected to predominate. Human longitudinal studies generally show little improvement in semiquantitative fatty-infiltration grade after repair (Gladstone et al., 2007; Melis et al., 2010). Selected neural-release and beige-fat experiments modify individual histological endpoints in animals (Lee et al., 2020c; Wang et al., 2021; Wang et al., 2020; Iio et al., 2023; Zhou et al., 2023; Wang et al., 2018b), but none establishes reversal of advanced human fibrosis or fibrofatty collapse.

## 6 Candidate two-dimensional research phenotyping and validation

A candidate research phenotype should begin with established structural variables and preserve quantitative measures as continuous data. Only after prospective validation should investigators explore separate probabilistic labels for dominant mechanism (mechanical-unloading, neural modifier, or mixed) and biological stage (Table 1). Standard MRI reporting should still include tear size, tendon involvement, retraction, Goutallier grade, tangent sign, and muscle volume, with regional fat distribution and quantitative measures added where available.

**TABLE 1:** Hypothesis-generating two-dimensional TMN research framework. The categories and candidate measures are unvalidated and should not be used for diagnosis, staging, prognosis, or treatment selection.

Dimension	Category	Working definition	Evidence calibration	Candidate measures and boundary
Dominant mechanism	Mechanical-unloading dominant	Tendon retraction and loss of load transfer predominate, with a concordant regional pattern of atrophy, fat, and impaired function. If the neural domain has not been assessed, the mechanism should remain indeterminate rather than be assigned by default	Human associative plus animal causal evidence	Retraction, tendon mobility, regional qMRI distribution, muscle volume, and strength. Absence of a neural modifier should not be inferred without appropriate assessment
Dominant mechanism	Neural modifier	An objectively measured, anatomically concordant peripheral or recruitment abnormality modifies the phenotype. Exploratory NMJ findings do not currently define this category	Direct human evidence exists for selected neuropathy; recruitment evidence is associative; cuff-specific NMJ evidence is limited and largely extrapolative	Clinical localization, EMG/NCS, nerve imaging, and prespecified recruitment measures. Pain-limited recruitment and NMJ research endpoints should be recorded separately
Dominant mechanism	Mixed	Mechanical unloading coexists with a defined or probable neural modifier	Supported by independent-effects animal models; prospective human clustering remains unvalidated	Integrated structural, electrophysiological and functional assessment. Avoid inferring synergy without longitudinal data
Biological stage	Early stress	Reduced tension or activation with edema-like/functional change but limited tissue replacement	Human associative imaging plus mechanistic hypothesis	T2-sensitive imaging, volume, activation and strength. Calendar time alone does not define stage
Biological stage	Intermediate remodeling	Measurable regional fat and/or collagen accumulation with residual contractile reserve; cellular and immune states are exploratory correlates rather than defining criteria	Animal causal and general muscle evidence; limited longitudinal human tissue evidence	Regional fat fraction, normalized or fat-free muscle volume, fibrosis-sensitive measures, strength, and contractile reserve; research tissue profiling is exploratory
Biological stage	Advanced fibrofatty replacement	Mature fat, collagen-rich fibrosis, severe atrophy and low contractile reserve dominate	Direct human imaging/histology and outcome association	Fat fraction, muscle volume, stiffness, mobility and objective function. Biological reversal should not be presumed

A mechanical-unloading-dominant assignment would require concordance between tear/retraction and the regional muscle pattern; if the neural domain has not been assessed, the mechanism should remain indeterminate rather than be assigned by default. A neural-modifier assignment would require an objectively measured, anatomically concordant peripheral or recruitment abnormality. Severe disease, pain-limited recruitment, or exploratory NMJ morphology alone would not define a mixed mechanism.

Electrodiagnostic testing is most informative when used selectively. EMG/NCS can support peripheral neuropathy or a motor-unit abnormality, but it neither measures pain-related central inhibition nor proves NMJ pathology. A normal study does not exclude mechanically driven fatty infiltration, and an abnormal study does not show that decompression alone will restore muscle quality.

Candidate stage should be studied independently using continuous imaging and function, with tissue profiling as an exploratory correlate. PDGFR-alpha-associated stromal populations, adipogenic and fibrotic readouts, macrophage states, mitochondrial markers, and NMJ morphology do not currently define a stage or routine clinical category.

### 6.1 Candidate operational definitions for prospective validation

Future studies should retain electrophysiological and functional measures as continuous variables in primary analyses. A candidate ‘electrodiagnostically supported neural modifier’ could require an anatomically localizing needle-EMG abnormality in the supraspinatus and/or infraspinatus—active denervation, chronic neurogenic motor-unit morphology, or reduced recruitment—together with a quantitative nerve-conduction abnormality relative to laboratory reference limits or the contralateral side. In a normal-subject study, a side-to-side latency difference of 0.4 m was proposed as an acceptable upper limit (Casazza et al., 1998); it should be treated only as an exploratory sensitivity anchor, not a cuff-specific diagnostic cutoff. Deltoid, cervical paraspinal, and other control muscles are needed to distinguish suprascapular neuropathy from radiculopathy, plexopathy, or generalized neuropathy. Pain-limited recruitment should be coded separately, and conventional EMG/NCS should not be interpreted as proof of NMJ pathology.

Quantitative MRI fat fraction should likewise remain continuous in primary analyses. In a prospective six-point Dixon cohort, supraspinatus fat fraction was explored using <8%, 8% to <13.5%, and ≥13.5% bands, with progression defined as an increase of at least 4.5 percentage points (Hochreiter et al., 2024). These cohort-derived values are not TMN-stage thresholds and should not be transferred across sequences, scanners, muscles, or populations without calibration. An advanced phenotype should require convergent evidence—high fat fraction together with reduced normalized or fat-free muscle volume and impaired strength or contractile reserve—rather than fat fraction alone. All categories require test–retest and interobserver reliability, internal derivation, external validation, and incremental value beyond established structural variables.

### 6.2 Modifiers and boundary conditions

Age, diabetes, obesity, sarcopenia, smoking, sex, tear chronicity, pain severity, and rehabilitation exposure can influence muscle volume, fat fraction, neural activation, inflammation, and healing. These are not merely background variables; they may shift both mechanism and stage and should be prespecified as covariates. Metabolic risk and general muscle health are particularly relevant when fat is diffuse or recovery is poor.

We restrict this model to chronic large/massive posterosuperior tears because supraspinatus/infraspinatus retraction, suprascapular nerve anatomy, and the available evidence on muscle degeneration intersect most clearly in this setting. Acute tears, isolated subscapularis or teres minor disease, revision repair, and cuff-tear arthropathy will require separate validation.

Figure 2 summarizes candidate temporal descriptors for prospective validation rather than established clinical stages. Table 2 situates the proposed TMN outputs against the emphases of prior review literature across five domains: structural prognosis, mechanics, neural biology, stromal biology, and validation.

**FIGURE 2:**
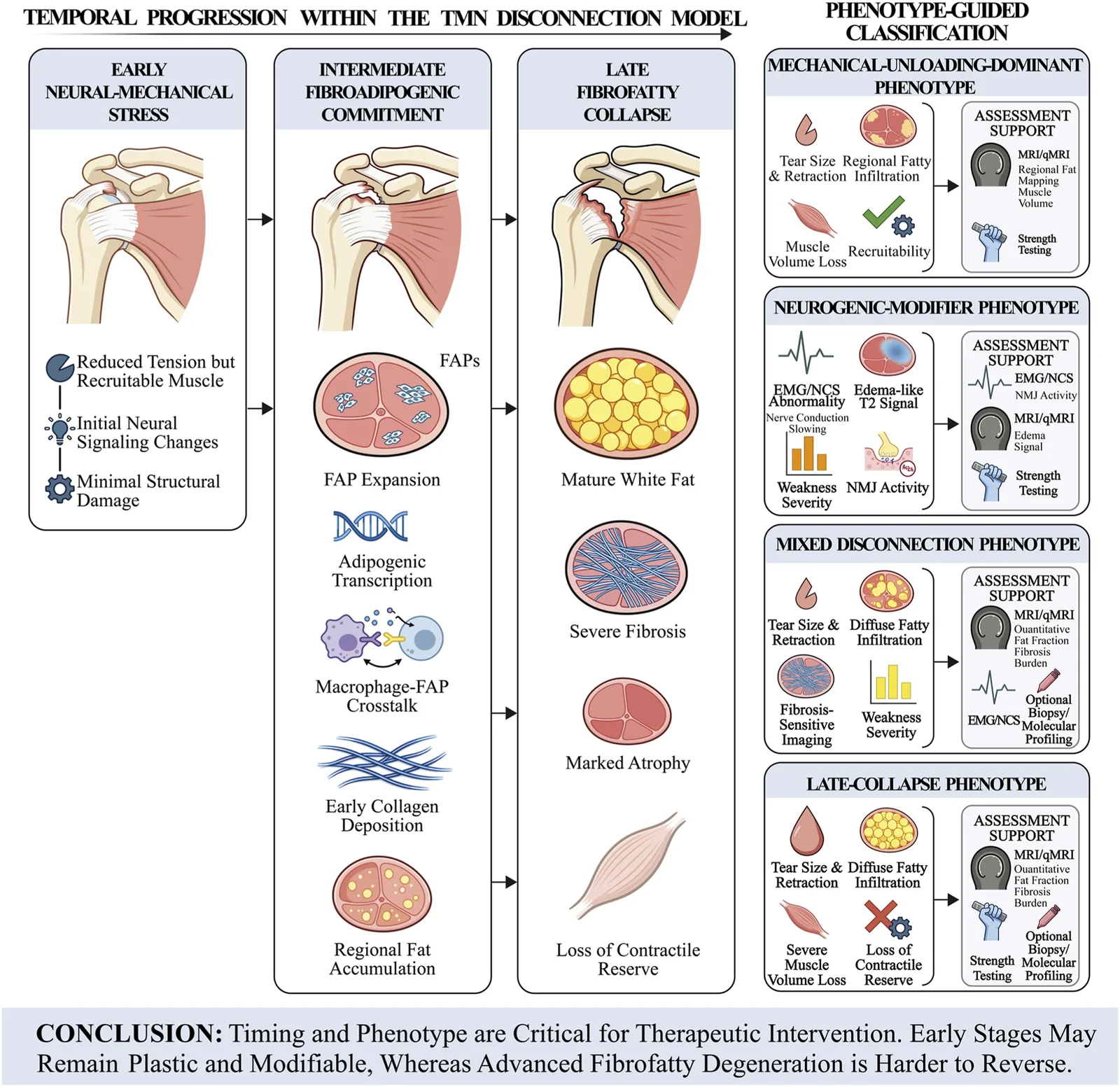
Unvalidated candidate biological trajectories within the TMN research framework. Early stress, intermediate remodeling, and advanced fibrofatty replacement are conceptual research descriptors without validated human TMN-stage cutoffs. The figure is not a validated clinical staging system, and dominant mechanism should be assessed independently.

**TABLE 2:** Contextual comparison of review domains and proposed research outputs; this table does not constitute a separate classification system.

Review domain	Common emphasis in prior literature	Addition in the TMN framework	Testable output
Structural prognosis	Tear size, retraction, fatty infiltration, atrophy and tendon healing	Separates structural continuity from contractile recruitability and useful force transmission	Incremental prediction of strength/endurance beyond structural variables
Mechanics	Tendon repair, footprint coverage and force couples	Treats loss and restoration of intramuscular-to-bone load transfer as a biological signal	Regional qMRI and function after controlled tension restoration
Neural biology	Suprascapular neuropathy considered as present or absent	Separates peripheral neuropathy, central inhibition, motor-unit recruitment and NMJ status	Component-specific electrophysiological and functional phenotyping
Stromal biology	Fatty infiltration as an imaging endpoint or isolated treatment target.	Links FAP state, immune signaling and ECM remodeling while grading the evidence source	Tissue-state markers paired with imaging and force
Time	Chronicity treated mainly as calendar duration	Defines biological stage independently from dominant mechanism	Mechanism-by-stage interaction and reversibility testing
Validation	Descriptive association or single-target intervention	Requires added prognostic value and prespecified falsification criteria	Nested models, calibration and prospective external validation

## 7 Translational hypotheses and clinical boundaries

Mechanical restoration remains the most firmly supported clinical principle. Tendon continuity, appropriate tension, tissue mobility, and the choice of reconstruction or augmentation determine whether a load-bearing muscle-tendon-bone circuit can be re-established. That clinical foundation should be kept distinct from unvalidated claims that established biological remodeling can be reversed.

Neural evaluation is useful when it is selective and component-specific. Documented suprascapular or spinoglenoid compression, traction neuropathy, and postoperative nerve injury may alter management, whereas pain-related inhibition, motor-unit under-recruitment, and NMJ remodeling require different measurements. Available evidence does not justify routine nerve release for all massive tears (Boykin et al., 2010; Mallon et al., 2006; Costouros et al., 2007; Lafosse et al., 2007; Collin et al., 2014; Gerber et al., 2020; Beeler et al., 2013; Leclere et al., 2014; Mistretta et al., 2025; Eshima et al., 2023; Wang et al., 2018b; Kong et al., 2016).

Rehabilitation is both a load-and-activation intervention and a postoperative protocol. Excessive early loading may jeopardize tendon healing, whereas prolonged underloading may deepen atrophy and loss of activation. Studies of protected motion, scapular motor control, graded isometrics, and progressive resistance should report repair integrity together with strength, endurance, and muscle quality.

Because apparent efficacy depends strongly on model architecture and treatment timing, candidate biological interventions should be read as experimental probes rather than a therapeutic menu. Imatinib and retinoic-acid-receptor agonists were tested from the early postoperative period in severe combined-injury models (Shirasawa et al., 2017; Shirasawa et al., 2021), and macrophage-derived exosomes were delivered at injury in a tendon-transection-plus-denervation model (Liu et al., 2023). These experiments primarily address prevention of early stromal commitment. By contrast, beige-FAP delivery at delayed repair, delayed beta3-adrenergic agonism, and liraglutide initiated 2 weeks after an unrepaired tear provide limited analogues of intermediate remodeling (Lee et al., 2020b; Wang et al., 2021; Yoon et al., 2026). Reported changes in histological fat, fibrosis, or muscle-related measures do not establish reversal of advanced disease, tendon-healing safety, or human efficacy. Table 3 therefore summarizes timing, model context, principal outcomes, and translational boundaries.

**TABLE 3:** Evidence-calibrated examples of clinical principles and preclinical interventions, including model context, timing, reported outcomes, and translational boundaries.

Strategy/target	Representative example	Model/timing and principal reported outcome	Framework placement and translational boundary
Mechanical restoration	Repair, tension restoration, reconstruction, or augmentation	Clinical and biomechanical evidence supports restoration of load transfer; muscle quality and tendon healing remain distinct outcomes	Clinical foundation across candidate stages, but not sufficient when tendon mobility or contractile reserve is poor
Selective neural management	Lesion-specific evaluation; decompression only for a documented, localizable lesion	Selected human observational/electrodiagnostic evidence and a mouse compression-release model; routine decompression is unsupported without a documented lesion (Collin et al., 2014; Gerber et al., 2020; Wang et al., 2018b)	Candidate early documented neural modifier; does not apply to pain inhibition or hypothesized NMJ remodeling and is usually adjunctive to mechanical restoration
Rehabilitation/load	Protected activation, scapular control, graded resistance, and research NMES	Clinical principle; timing and dose should be studied with repair integrity, strength, endurance, and muscle-quality outcomes	Candidate activity-maintenance adjunct; balance tendon protection against prolonged underloading and do not infer NMJ rescue
Stromal/ECM perturbation	Imatinib (PDGFR-linked) (Shirasawa et al., 2017); retinoic-acid-receptor agonists (Shirasawa et al., 2021)	Severe mouse combined injury with tendon transection, denervation, and humeral-head resection. Imatinib 30 mg/kg orally, 5 days/week, was assessed at 2–4 weeks; RAR agonists were initiated after surgery and assessed at 4 weeks. Both reduced adipogenic markers and histological fat; RAR agonism increased collagen transcripts without histological collagen accumulation	Candidate early-stress/prevention experiments. Target specificity, dose, delivery, systemic safety, fibrosis, and tendon-healing effects are unresolved; these are not clinical recommendations
Macrophage/EV niche	M0/M1/M2 macrophage-derived exosomes (Liu et al., 2023)	Mouse supraspinatus/infraspinatus transection plus denervation; 2 × 10^9 exosomes were injected into supraspinatus at injury and assessed at 6 weeks. M2-derived exosomes were associated with less fat, fibrosis, and atrophy and with greater UCP1-related beige differentiation	Small, single prophylactic model that tests early niche modulation, not established-disease reversal. EV identity, manufacturing, biodistribution, durability, and safety remain unresolved
Beige/metabolic fate	BAT-FAP delivery (Lee et al., 2020b); amibegron (Wang et al., 2021); liraglutide (Yoon et al., 2026)	BAT-FAPs (250,000 cells) were delivered at 2- or 6-week delayed repair with 6-week follow-up in mice; amibegron 10 mg/kg/day was started immediately or at week 6 for 6 weeks in a mouse transection-plus-denervation model; liraglutide 250 μg/kg/day intraperitoneally was started 2 weeks after an unrepaired rat supraspinatus tear for 4 weeks. Selected studies reported less histological fat/atrophy and partial structural, gait, ROM, or CMAP improvement	Delayed models provide limited intermediate-remodeling analogues, but none demonstrates advanced human reversal or clinical efficacy. Systemic effects, durability, tendon repair, and safety require testing; CMAP does not prove NMJ repair
Advanced-stage compensation	Tendon transfer, reconstruction, arthroplasty, and compensation-focused rehabilitation	Established clinical options for selected patients, chosen using conventional indications rather than the unvalidated TMN framework	Candidate advanced-stage goal: compensate for irreversible loss rather than presume biological reversal

Advanced fibrofatty replacement requires realistic goals. When tendon mobility and contractile reserve are poor, salvage reconstruction, tendon transfer, superior capsular reconstruction, reverse shoulder arthroplasty, or compensation-focused rehabilitation may be considered according to established clinical indications. The TMN framework is not validated to select among these options.

## 8 Framework validation and future research

Validation should proceed in sequence: measurement reliability, prospective phenotype derivation, incremental prognosis beyond established structural variables, external validation, and only then treatment-effect modification. Mechanism and candidate stage should remain separate variables rather than being collapsed into another severity score.

Prospective cohorts should integrate qMRI fat fraction and regional distribution, muscle volume, edema-like or altered T2 signal, component-specific neural measures, objective strength and endurance, pain and motor-control measures, tendon integrity, metabolic risk, and rehabilitation exposure. Age, sex, diabetes, obesity/sarcopenia, smoking, and chronicity need to be prespecified covariates rather than *post hoc* explanations.

A minimum dataset makes the proposal falsifiable. Mechanism and stage assignments must be reproducible and must improve calibration, discrimination, or the detection of treatment-effect heterogeneity beyond tear size, retraction, Goutallier grade, and tendon healing alone.

Preclinical designs should distinguish initiating injuries from downstream pathways by including sham, isolated tendon injury, isolated nerve injury, combined injury, delayed repair, and nerve-release groups. Sampling across biological stages should incorporate sex, age, and metabolic context. Supplementary Table S3, provided in the separate Supplementary Material, summarizes the proposed experimental matrix.

Intervention studies could test whether adding a mechanism- and stage-matched adjunct to mechanical restoration improves outcomes. Early experiments should distinguish prevention after disruption of loading or activation from reversal after established remodeling; delayed and repaired models are needed before clinical translation. Relevant outcomes include tendon integrity, objective force, endurance, qMRI fat fraction and muscle volume, fibrosis, and patient-reported function (Zbinden et al., 2026; Lebaschi et al., 2016).

Clinical use would require prospective external validation and evidence that the framework improves decisions or outcomes; the present model is a research agenda only.

Figure 3 summarizes experimental research questions and should not be interpreted as a therapeutic algorithm (Figure 3 should be placed here).

Box 1 **| Testable hypotheses and minimum validation logic generated by the proposed model. These five hypotheses (H1–H5) form a sequential validation agenda: confirming the reproducibility of the two core dimensions (H1) is a prerequisite for testing the mechanism-by-stage and biomarker incremental-value hypotheses (H2–H4), which in turn precede evaluation of combined intervention strategies (H5). None of these hypotheses has yet been tested; they specify what prospective studies would need to demonstrate before the TMN framework could inform clinical decisions.**

H1: Are dominant mechanism and candidate biological stage reproducible as separate dimensions?

H2: Do peripheral neuropathy, pain-related central inhibition, motor-unit recruitment, and exploratory NMJ measures show distinct associations with regional fat, weakness, and recovery?

H3: Does a mechanism-by-stage interaction improve prediction beyond established structural variables?

H4: Do FAP/ECM and macrophage-state measures add incremental prognostic information beyond qMRI after adjustment for prespecified confounders?

H5: In validated intermediate-stage models, does combined mechanical restoration and biologic fate modulation outperform either strategy alone without compromising tendon healing or safety?

**FIGURE 3:**
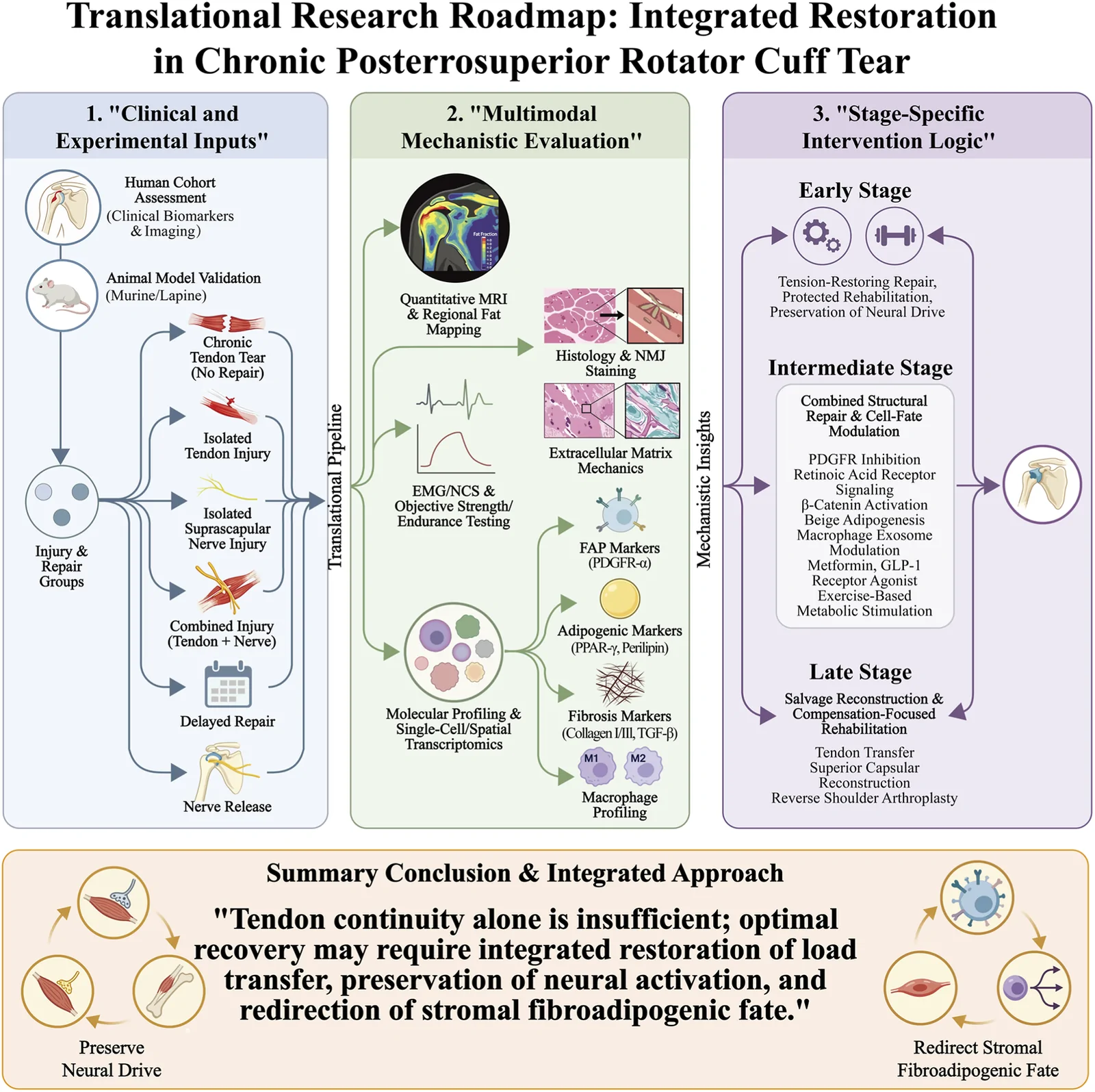
Hypothesis-generating translational research agenda for chronic rotator cuff muscle dysfunction. Molecular, cellular, neural, and metabolic interventions shown in the figure are predominantly preclinical or extrapolative and should not be interpreted as treatment recommendations, efficacy rankings, or a clinical management algorithm.

## 9 Limitations and interpretive boundaries

The searches were structured and documented, but relevance-based selection was not independently duplicated and no formal risk-of-bias assessment was performed. Omission and selection bias therefore remain possible, and the English-language restriction may have excluded relevant evidence. The evidence-provenance categories are interpretive aids rather than formal certainty grades.

Human evidence is strongest for the prognostic relevance of imaging-defined fatty infiltration, atrophy, and muscle volume. Human cuff evidence for fibrosis is less mature and often cross-sectional. Cuff-specific evidence remains limited for NMJ remodeling, pain-related activation deficits, longitudinal FAP states, macrophage–extracellular-vesicle signaling, and biological reversibility. Animal perturbation and non-cuff skeletal-muscle studies support plausibility but cannot establish mechanism or treatment efficacy in patients.

Neither the proposed electrophysiological designation nor the qMRI anchors has been validated for TMN diagnosis, staging, prognosis, reproducibility, or treatment selection. Prospective studies should report variables continuously, prespecify exploratory categories, assess test–retest and interobserver reliability, and perform internal and external validation. Most biological interventions were tested in young rodents using acute or severe combined injury; changes in adipogenic markers or histological fat should not be equated with durable force recovery, tendon healing, or reversal of chronic human disease.

The treatment discussion is therefore a map of experimental questions, not clinical recommendations. Translation requires human safety, dose, delivery, target engagement, durability, and efficacy data, as well as proof that any adjunct does not compromise tendon-to-bone healing.

## 10 Conclusion

Chronic large and massive posterosuperior rotator cuff tears cannot be explained by tendon discontinuity alone, but their biology should not be compressed into a single disconnection phenotype. The proposed TMN framework separates dominant mechanism from candidate biological stage, distinguishes objectively measurable neural components, and treats possible NMJ remodeling as a hypothesis-level domain. Its value depends on prospective demonstration of reproducibility, incremental prediction, external validity, and treatment-effect modification beyond tear morphology, muscle-quality imaging, and tendon healing. Mechanical restoration remains the clinical foundation; neural evaluation is selective, and stromal or molecular interventions remain preclinical research hypotheses.

## Author’s note

This article is a targeted narrative mechanistic review and hypothesis-generating research framework rather than a systematic review. Structured, targeted searches of PubMed, Scopus, and Web of Science were conducted from database inception through 30 May 2026, restricted to English-language records, and supplemented by backward and forward citation tracking. Database-specific search strings, eligibility criteria, and the relevance-based selection process are provided in Supplementary Table S1. Human imaging, outcome, electrodiagnostic, and histological studies were prioritized for cuff-specific clinical observations; rotator cuff animal models were used to examine perturbational mechanisms within the model studied; and non-cuff skeletal-muscle studies were included only when they directly informed NMJ, FAP, immune, or ECM biology that was not adequately resolved by cuff-specific evidence. For each mechanistic claim, we distinguished direct measurement in human cuff disease, human cuff association, controlled perturbation in a cuff-specific animal model, mechanistic evidence from non-cuff skeletal muscle, and an untested hypothesis. These categories describe evidence provenance rather than a formal certainty hierarchy; ‘causal’ refers only to effects demonstrated within the tested animal model. When findings diverged, differences in population and tear definition, chronicity, injury model, intervention timing, assay, sample size, and major confounders were considered, and discordance was reported rather than resolved by study count. Study selection was not independently duplicated, and no formal risk-of-bias assessment, PRISMA flow diagram, or meta-analysis was performed. Accordingly, the corpus should be interpreted as targeted and relevance-based rather than exhaustive; a SANRA-informed self-audit is provided in Supplementary Table S2.

## References

[B1] Agha O. Diaz A. Davies M. Kim H. T. Liu X. Feeley B. T. (2021). Rotator cuff tear degeneration and the role of fibro-adipogenic progenitors. Ann. N. Y. Acad. Sci. 1490 (1), 13–28. 10.1111/nyas.14437 32725671 PMC7855559

[B2] Arnold L. Henry A. Poron F. Baba-Amer Y. van Rooijen N. Plonquet A. (2007). Inflammatory monocytes recruited after skeletal muscle injury switch into antiinflammatory macrophages to support myogenesis. J. Exp. Med. 204 (5), 1057–1069. 10.1084/jem.20070075 17485518 PMC2118577

[B3] Beeler S. Ek E. T. Gerber C. (2013). A comparative analysis of fatty infiltration and muscle atrophy in patients with chronic rotator cuff tears and suprascapular neuropathy. J. Shoulder Elb. Surg. 22 (11), 1537–1546. 10.1016/j.jse.2013.01.028 23642348

[B4] Bentzinger C. F. Wang Y. X. Rudnicki M. A. (2012). Building muscle: molecular regulation of myogenesis. Cold Spring Harb. Perspect. Biol. 4 (2), a008342. 10.1101/cshperspect.a008342 22300977 PMC3281568

[B5] Biferali B. Proietti D. Mozzetta C. Madaro L. (2019). Fibro-adipogenic progenitors cross-talk in skeletal muscle: the social network. Front. Physiol. 10, 1074. 10.3389/fphys.2019.01074 31496956 PMC6713247

[B6] Blaauw B. Schiaffino S. Reggiani C. (2013). Mechanisms modulating skeletal muscle phenotype. Compr. Physiol. 3 (4), 1645–1687. 10.1002/cphy.c130009 24265241

[B7] Bodine S. C. Stitt T. N. Gonzalez M. Kline W. O. Stover G. L. Bauerlein R. (2001). Akt/mTOR pathway is a crucial regulator of skeletal muscle hypertrophy and can prevent muscle atrophy *in vivo* . Nat. Cell Biol. 3 (11), 1014–1019. 10.1038/ncb1101-1014 11715023

[B8] Bogdanov J. Lan R. Chu T. N. Bolia I. K. Weber A. E. Petrigliano F. A. (2021). Fatty degeneration of the rotator cuff: pathogenesis, clinical implications, and future treatment. JSES Rev. Rep. Tech. 1 (4), 301–308. 10.1016/j.xrrt.2021.05.006 37588720 PMC10426606

[B9] Bonaldo P. Sandri M. (2013). Cellular and molecular mechanisms of muscle atrophy. Dis. Model Mech. 6 (1), 25–39. 10.1242/dmm.010389 23268536 PMC3529336

[B10] Bowen E. Waque A. Su F. Davies M. Ode G. Lansdown D. (2025). Muscle health and fatty infiltration with advanced rotator cuff pathology. Curr. Rev. Musculoskelet. Med. 18 (4), 160–172. 10.1007/s12178-025-09955-w 40009348 PMC11965080

[B11] Boykin R. E. Friedman D. J. Higgins L. D. Warner J. J. (2010). Suprascapular neuropathy. J. Bone Jt. Surg. Am. 92 (13), 2348–2364. 10.2106/JBJS.I.01743 20926731

[B12] Burkhart S. S. (1997). Partial repair of massive rotator cuff tears: the evolution of a concept. Orthop. Clin. North Am. 28 (1), 125–132. 10.1016/s0030-5898(05)70270-4 9024437

[B13] Burkhart S. S. Esch J. C. Jolson R. S. (1993). The rotator crescent and rotator cable: an anatomic description of the shoulder’s suspension bridge. Arthroscopy 9 (6), 611–616. 10.1016/s0749-8063(05)80496-7 8305096

[B14] Burkhart S. S. Morgan C. D. Kibler W. B. (2003). The disabled throwing shoulder: spectrum of pathology part I: pathoanatomy and biomechanics. Arthroscopy 19 (4), 404–420. 10.1053/jars.2003.50128 12671624

[B15] Casazza B. A. Young J. L. Press J. P. Heinemann A. W. (1998). Suprascapular nerve conduction: a comparative analysis in normal subjects. Electromyogr. Clin. Neurophysiol. 38 (3), 153–160. 9637941

[B16] Chazaud B. (2014). Macrophages: supportive cells for tissue repair and regeneration. Immunobiology 219 (3), 172–178. 10.1016/j.imbio.2013.09.001 24080029

[B17] Collin P. Treseder T. Lädermann A. Benkalfate T. Mourtada R. Courage O. (2014). Neuropathy of the suprascapular nerve and massive rotator cuff tears: a prospective electromyographic study. J. Shoulder Elb. Surg. 23 (1), 28–34. 10.1016/j.jse.2013.07.039 24090983

[B18] Contreras O. Rossi F. M. V. Theret M. (2021). Origins, potency, and heterogeneity of skeletal muscle fibro-adipogenic progenitors-time for new definitions. Skelet. Muscle 11 (1), 16. 10.1186/s13395-021-00265-6 34210364 PMC8247239

[B19] Costouros J. G. Porramatikul M. Lie D. T. Warner J. J. (2007). Reversal of suprascapular neuropathy following arthroscopic repair of massive supraspinatus and infraspinatus rotator cuff tears. Arthroscopy 23 (11), 1152–1161. 10.1016/j.arthro.2007.06.014 17986401

[B20] Davies M. R. Chi H. Kaur G. Liu M. Ma C. B. Kim H. T. (2022). Rotator cuff tear size regulates fibroadipogenic progenitor number and gene expression profile in the supraspinatus independent of patient age. Am. J. Sports Med. 50 (1), 208–215. 10.1177/03635465211054512 34779676 PMC9280916

[B21] Egerman M. A. Glass D. J. (2014). Signaling pathways controlling skeletal muscle mass. Crit. Rev. Biochem. Mol. Biol. 49 (1), 59–68. 10.3109/10409238.2013.857291 24237131 PMC3913083

[B22] Eshima K. Ohzono H. Gotoh M. Shimokobe H. Tanaka K. Nakamura H. (2023). Effect of suprascapular nerve injury on muscle and regenerated enthesis in a rat rotator cuff tear model. Clin. Shoulder Elb. 26 (2), 131–139. 10.5397/cise.2022.01207 37316174 PMC10277713

[B23] Flores-Opazo M. Kopinke D. Helmbacher F. Fernández-Verdejo R. Tuñón-Suárez M. Lynch G. S. (2024). Fibro-adipogenic progenitors in physiological adipogenesis and intermuscular adipose tissue remodeling. Mol. Asp. Med. 97, 101277. 10.1016/j.mam.2024.101277 38788527 PMC11692456

[B24] Fuchs B. Weishaupt D. Zanetti M. Hodler J. Gerber C. (1999). Fatty degeneration of the muscles of the rotator cuff: assessment by computed tomography versus magnetic resonance imaging. J. Shoulder Elb. Surg. 8 (6), 599–605. 10.1016/s1058-2746(99)90097-6 10633896

[B25] Galatz L. M. Ball C. M. Teefey S. A. Middleton W. D. Yamaguchi K. (2004). The outcome and repair integrity of completely arthroscopically repaired large and massive rotator cuff tears. J. Bone Jt. Surg. Am. 86 (2), 219–224. 10.2106/00004623-200402000-00002 14960664

[B26] Gerber C. Fuchs B. Hodler J. (2000). The results of repair of massive tears of the rotator cuff. J. Bone Jt. Surg. Am. 82 (4), 505–515. 10.2106/00004623-200004000-00006 10761941

[B27] Gerber C. Meyer D. C. Schneeberger A. G. Hoppeler H. von Rechenberg B. (2004). Effect of tendon release and delayed repair on the structure of the muscles of the rotator cuff: an experimental study in sheep. J. Bone Jt. Surg. Am. 86 (9), 1973–1982. 10.2106/00004623-200409000-00016 15342760

[B28] Gerber C. Schneeberger A. G. Hoppeler H. Meyer D. C. (2007). Correlation of atrophy and fatty infiltration on strength and integrity of rotator cuff repairs: a study in thirteen patients. J. Shoulder Elb. Surg. 16 (6), 691–696. 10.1016/j.jse.2007.02.122 17931904

[B29] Gerber C. Meyer D. C. Wieser K. Sutter R. Schubert M. Kriechling P. (2020). Suprascapular nerve decompression in addition to rotator cuff repair: a prospective, randomized observational trial. J. Shoulder Elb. Surg. 29 (8), 1633–1641. 10.1016/j.jse.2020.03.051 32713467

[B30] Gibbons M. C. Singh A. Anakwenze O. Cheng T. Pomerantz M. Schenk S. (2017). Histological evidence of muscle degeneration in advanced human rotator cuff disease. J. Bone Jt. Surg. Am. 99 (3), 190–199. 10.2106/JBJS.16.00335 28145949 PMC5395080

[B31] Gillies A. R. Lieber R. L. (2011). Structure and function of the skeletal muscle extracellular matrix. Muscle Nerve 44 (3), 318–331. 10.1002/mus.22094 21949456 PMC3177172

[B32] Gladstone J. N. Bishop J. Y. Lo I. K. Flatow E. L. (2007). Fatty infiltration and atrophy of the rotator cuff do not improve after rotator cuff repair and correlate with poor functional outcome. Am. J. Sports Med. 35 (5), 719–728. 10.1177/0363546506297539 17337727

[B33] Goutallier D. Postel J. M. Bernageau J. Lavau L. Voisin M. C. (1994). Fatty muscle degeneration in cuff ruptures. Pre- and postoperative evaluation by CT scan. Clin. Orthop. Relat. Res. 304, 78–83. 8020238

[B34] Goutallier D. Postel J. M. Gleyze P. Leguilloux P. Van Driessche S. (2003). Influence of cuff muscle fatty degeneration on anatomic and functional outcomes after simple suture of full-thickness tears. J. Shoulder Elb. Surg. 12 (6), 550–554. 10.1016/s1058-2746(03)00211-8 14671517

[B35] Greenberg A. S. Reeves A. R. (2021). The good and bad of adipose tissue macrophage exosomes in obesity. Cell Metab. 33 (4), 700–702. 10.1016/j.cmet.2021.03.011 33826911

[B36] Gumucio J. P. Korn M. A. Saripalli A. L. Flood M. D. Phan A. C. Roche S. M. (2014). Aging-associated exacerbation in fatty degeneration and infiltration after rotator cuff tear. J. Shoulder Elb. Surg. 23 (1), 99–108. 10.1016/j.jse.2013.04.011 23790676 PMC3785561

[B37] Harryman D. T. Mack L. A. Wang K. Y. Jackins S. E. Richardson M. L. Matsen F. A. (1991). 3rd: Reprirs of the rotator cuff. Correlation of functional results with integrity of the cuff. J. Bone Jt. Surg. Am. 73 (7), 982–989. 10.2106/00004623-199173070-00004 1874784

[B38] Heredia J. E. Mukundan L. Chen F. M. Mueller A. A. Deo R. C. Locksley R. M. (2013). Type 2 innate signals stimulate fibro/adipogenic progenitors to facilitate muscle regeneration. Cell 153 (2), 376–388. 10.1016/j.cell.2013.02.053 23582327 PMC3663598

[B39] Hochreiter B. Germann C. Feuerriegel G. C. Sutter R. Selman F. Gressl M. (2024). Natural history of quantitative fatty infiltration and 3D muscle volume after nonoperative treatment of symptomatic rotator cuff tears: a prospective MRI study of 79 patients. J. Bone Jt. Surg. Am. 106 (8), 690–699. 10.2106/jbjs.23.01083 38386719 PMC12341752

[B40] Iio R. Manaka T. Takada N. Orita K. Nakazawa K. Hirakawa Y. (2023). Parathyroid hormone inhibits fatty infiltration and muscle atrophy after rotator cuff tear by browning of fibroadipogenic progenitors in a rodent model. Am. J. Sports Med. 51 (12), 3251–3260. 10.1177/03635465231190389 37621014

[B41] Joe A. W. Yi L. Natarajan A. Le Grand F. So L. Wang J. (2010). Muscle injury activates resident fibro/adipogenic progenitors that facilitate myogenesis. Nat. Cell Biol. 12 (2), 153–163. 10.1038/ncb2015 20081841 PMC4580288

[B42] Jorgenson K. W. Phillips S. M. Hornberger T. A. (2020). Identifying the structural adaptations that drive the mechanical load-induced growth of skeletal muscle: a scoping review. Cells 9 (7), 1658. 10.3390/cells9071658 32660165 PMC7408414

[B43] Joshi S. K. Liu X. Samagh S. P. Lovett D. H. Bodine S. C. Kim H. T. (2013). mTOR regulates fatty infiltration through SREBP-1 and PPARγ after a combined massive rotator cuff tear and suprascapular nerve injury in rats. J. Orthop. Res. 31 (5), 724–730. 10.1002/jor.22254 23239524 PMC4393845

[B44] Keener J. D. Galatz L. M. Teefey S. A. Middleton W. D. Steger-May K. Stobbs-Cucchi G. (2015). A prospective evaluation of survivorship of asymptomatic degenerative rotator cuff tears. J. Bone Jt. Surg. Am. 97 (2), 89–98. 10.2106/JBJS.N.00099 25609434 PMC4296477

[B45] Kim H. M. Galatz L. M. Lim C. Havlioglu N. Thomopoulos S. (2012). The effect of tear size and nerve injury on rotator cuff muscle fatty degeneration in a rodent animal model. J. Shoulder Elb. Surg. 21 (7), 847–858. 10.1016/j.jse.2011.05.004 21831663 PMC3217129

[B46] Kong B. Y. Kim S. H. Kim D. H. Joung H. Y. Jang Y. H. Oh J. H. (2016). Suprascapular neuropathy in massive rotator cuff tears with severe fatty degeneration in the infraspinatus muscle. Bone Jt. J. 98-b (11), 1505–1509. 10.1302/0301-620X.98B11.37928 27803226

[B47] Kuzel B. R. Grindel S. Papandrea R. Ziegler D. (2013). Fatty infiltration and rotator cuff atrophy. J. Am. Acad. Orthop. Surg. 21 (10), 613–623. 10.5435/JAAOS-21-10-613 24084435

[B48] Lädermann A. Burkhart S. S. Hoffmeyer P. Neyton L. Collin P. Yates E. (2016). Classification of full-thickness rotator cuff lesions: a review. EFORT Open Rev. 1 (12), 420–430. 10.1302/2058-5241.1.160005 28461921 PMC5367545

[B49] Lafosse L. Tomasi A. Corbett S. Baier G. Willems K. Gobezie R. (2007). Arthroscopic release of suprascapular nerve entrapment at the suprascapular notch: technique and preliminary results. Arthroscopy 23 (1), 34–42. 10.1016/j.arthro.2006.10.003 17210425

[B50] Lebaschi A. Deng X. H. Zong J. Cong G. T. Carballo C. B. Album Z. M. (2016). Animal models for rotator cuff repair. Ann. N. Y. Acad. Sci. 1383 (1), 43–57. 10.1111/nyas.13203 27723933

[B51] Leclere L. E. Shi L. L. Lin A. Yannopoulos P. Higgins L. D. Warner J. J. (2014). Complete fatty infiltration of intact rotator cuffs caused by suprascapular neuropathy. Arthroscopy 30 (5), 639–644. 10.1016/j.arthro.2014.01.010 24630957

[B52] Lee C. Agha O. Liu M. Davies M. Bertoy L. Kim H. T. (2020a). Rotator cuff fibro-adipogenic progenitors demonstrate highest concentration, proliferative capacity, and adipogenic potential across muscle groups. J. Orthop. Res. 38 (5), 1113–1121. 10.1002/jor.24550 31799698 PMC9262119

[B53] Lee C. Liu M. Agha O. Kim H. T. Liu X. Feeley B. T. (2020b). Beige fibro-adipogenic progenitor transplantation reduces muscle degeneration and improves function in a mouse model of delayed repair of rotator cuff tears. J. Shoulder Elb. Surg. 29 (4), 719–727. 10.1016/j.jse.2019.09.021 31784382 PMC7085983

[B54] Lee C. Liu M. Agha O. Kim H. T. Feeley B. T. Liu X. (2020c). Beige FAPs transplantation improves muscle quality and shoulder function after massive rotator cuff tears. J. Orthop. Res. 38 (5), 1159–1166. 10.1002/jor.24558 31808573 PMC7162719

[B55] Lemos D. R. Babaeijandaghi F. Low M. Chang C. K. Lee S. T. Fiore D. (2015). Nilotinib reduces muscle fibrosis in chronic muscle injury by promoting TNF-Mediated apoptosis of fibro/adipogenic progenitors. Nat. Med. 21 (7), 786–794. 10.1038/nm.3869 26053624

[B56] Li L. Xiong W. C. Mei L. (2018). Neuromuscular junction formation, aging, and disorders. Annu. Rev. Physiol. 80, 159–188. 10.1146/annurev-physiol-022516-034255 29195055

[B57] Lieber R. L. Ward S. R. (2013). Cellular mechanisms of tissue fibrosis. 4. Structural and functional consequences of skeletal muscle fibrosis. Am. J. Physiol. Cell Physiol. 305 (3), C241–C252. 10.1152/ajpcell.00173.2013 23761627 PMC3742845

[B58] Lin X. Wang P. Wang W. Zhou H. Zhu S. Feng S. (2023). Suppressed Akt/GSK-3β/β-catenin signaling contributes to excessive adipogenesis of fibro-adipogenic progenitors after rotator cuff tears. Cell Death Discov. 9 (1), 312. 10.1038/s41420-023-01618-4 37626040 PMC10457376

[B59] Liu X. Manzano G. Kim H. T. Feeley B. T. (2011). A rat model of massive rotator cuff tears. J. Orthop. Res. 29 (4), 588–595. 10.1002/jor.21266 20949443

[B60] Liu X. Laron D. Natsuhara K. Manzano G. Kim H. T. Feeley B. T. (2012). A mouse model of massive rotator cuff tears. J. Bone Jt. Surg. Am. 94 (7), e41. 10.2106/JBJS.K.00620 22488625

[B61] Liu X. Ning A. Y. Chang N. C. Kim H. Nissenson R. Wang L. (2016). Investigating the cellular origin of rotator cuff muscle fatty infiltration and fibrosis after injury. Muscles Ligaments Tendons J. 6 (1), 6–15. 10.11138/mltj/2016.6.1.006 27331027 PMC4915463

[B62] Liu Q. Tang Q. Liao L. Li D. Zhu W. Zhao C. (2022). Translational therapy from preclinical animal models for muscle degeneration after rotator cuff injury. J. Orthop. Transl. 35, 13–22. 10.1016/j.jot.2022.03.002 35846726 PMC9260436

[B63] Liu M. Ng M. Phu T. Bouchareychas L. Feeley B. T. Kim H. T. (2023). Polarized macrophages regulate fibro/adipogenic progenitor (FAP) adipogenesis through exosomes. Stem Cell Res. Ther. 14 (1), 321. 10.1186/s13287-023-03555-6 37936229 PMC10631219

[B64] Luan T. Liu X. Easley J. T. Ravishankar B. Puttlitz C. Feeley B. T. (2015). Muscle atrophy and fatty infiltration after an acute rotator cuff repair in a sheep model. Muscles Ligaments Tendons J. 5 (2), 106–112. 10.11138/mltj/2015.5.2.106 26261789 PMC4496009

[B65] Madaro L. Passafaro M. Sala D. Etxaniz U. Lugarini F. Proietti D. (2018). Denervation-activated STAT3-IL-6 signalling in fibro-adipogenic progenitors promotes myofibres atrophy and fibrosis. Nat. Cell Biol. 20 (8), 917–927. 10.1038/s41556-018-0151-y 30050118 PMC6145844

[B66] Mall N. A. Kim H. M. Keener J. D. Steger-May K. Teefey S. A. Middleton W. D. (2010). Symptomatic progression of asymptomatic rotator cuff tears: a prospective study of clinical and sonographic variables. J. Bone Jt. Surg. Am. 92 (16), 2623–2633. 10.2106/JBJS.I.00506 21084574 PMC2970889

[B67] Mallon W. J. Wilson R. J. Basamania C. J. (2006). The association of suprascapular neuropathy with massive rotator cuff tears: a preliminary report. J. Shoulder Elb. Surg. 15 (4), 395–398. 10.1016/j.jse.2005.10.019 16831639

[B68] Mann C. J. Perdiguero E. Kharraz Y. Aguilar S. Pessina P. Serrano A. L. (2011). Aberrant repair and fibrosis development in skeletal muscle. Skelet. Muscle 1 (1), 21. 10.1186/2044-5040-1-21 21798099 PMC3156644

[B69] Melis B. DeFranco M. J. Chuinard C. Walch G. (2010). Natural history of fatty infiltration and atrophy of the supraspinatus muscle in rotator cuff tears. Clin. Orthop. Relat. Res. 468 (6), 1498–1505. 10.1007/s11999-009-1207-x 20094853 PMC2865597

[B70] Meyer D. C. Hoppeler H. von Rechenberg B. Gerber C. (2004). A pathomechanical concept explains muscle loss and fatty muscular changes following surgical tendon release. J. Orthop. Res. 22 (5), 1004–1007. 10.1016/j.orthres.2004.02.009 15304272

[B71] Mistretta K. L. Klein A. Lai J. K. Valencia A. P. Davis D. L. Williams K. (2025). The independent effect of nerve injury and tendon injury on rotator cuff fatty infiltration. Am. J. Sports Med. 53 (14), 3498–3505. 10.1177/03635465251391431 41243437

[B72] Molina T. Fabre P. Dumont N. A. (2021). Fibro-adipogenic progenitors in skeletal muscle homeostasis, regeneration and diseases. Open Biol. 11 (12), 210110. 10.1098/rsob.210110 34875199 PMC8651418

[B73] Mozzetta C. Consalvi S. Saccone V. Tierney M. Diamantini A. Mitchell K. J. (2013). Fibroadipogenic progenitors mediate the ability of HDAC inhibitors to promote regeneration in dystrophic muscles of young, but not old mdx mice. EMBO Mol. Med. 5 (4), 626–639. 10.1002/emmm.201202096 23505062 PMC3628105

[B74] Nakagaki K. Ozaki J. Tomita Y. Tamai S. (1995). Function of supraspinatus muscle with torn cuff evaluated by magnetic resonance imaging. Clin. Orthop. Relat. Res. 318, 144–151. 7671508

[B75] Negroni E. Kondili M. Muraine L. Bensalah M. Butler-Browne G. S. Mouly V. (2022). Muscle fibro-adipogenic progenitors from a single-cell perspective: focus on their “virtual” secretome. Front. Cell Dev. Biol. 10, 952041. 10.3389/fcell.2022.952041 36200044 PMC9527288

[B76] Patte D. (1990). Classification of rotator cuff lesions. Clin. Orthop. Relat. Res. 254, 81–86. 10.1097/00003086-199005000-00012 2323151

[B77] Rudolf R. Khan M. M. Labeit S. Deschenes M. R. (2014). Degeneration of neuromuscular junction in age and dystrophy. Front. Aging Neurosci. 6, 99. 10.3389/fnagi.2014.00099 24904412 PMC4033055

[B78] Rodeo S. A. (2007). Biologic augmentation of rotator cuff tendon repair. J. Shoulder Elb. Surg. 16 (5 Suppl. l), S191–S197. 10.1016/j.jse.2007.03.012 17574875

[B79] Rowshan K. Hadley S. Pham K. Caiozzo V. Lee T. Q. Gupta R. (2010). Development of fatty atrophy after neurologic and rotator cuff injuries in an animal model of rotator cuff pathology. J. Bone Jt. Surg. Am. 92 (13), 2270–2278. 10.2106/JBJS.I.00812 20926720 PMC2945930

[B80] Safran O. Schroeder J. Bloom R. Weil Y. Milgrom C. (2011). Natural history of nonoperatively treated symptomatic rotator cuff tears in patients 60 years old or younger. Am. J. Sports Med. 39 (4), 710–714. 10.1177/0363546510393944 21310940

[B81] Sandri M. (2013). Protein breakdown in muscle wasting: role of autophagy-lysosome and ubiquitin-proteasome. Int. J. Biochem. Cell Biol. 45 (10), 2121–2129. 10.1016/j.biocel.2013.04.023 23665154 PMC3775123

[B82] Sandri M. Sandri C. Gilbert A. Skurk C. Calabria E. Picard A. (2004). Foxo transcription factors induce the atrophy-related ubiquitin ligase atrogin-1 and cause skeletal muscle atrophy. Cell 117 (3), 399–412. 10.1016/s0092-8674(04)00400-3 15109499 PMC3619734

[B83] Santago A. C. Vidt M. E. Tuohy C. J. Poehling G. G. Freehill M. T. Jordan J. H. (2016). Quantitative analysis of three-dimensional distribution and clustering of intramuscular fat in muscles of the rotator cuff. Ann. Biomed. Eng. 44 (7), 2158–2167. 10.1007/s10439-015-1488-z 26514349 PMC4851609

[B84] Schiaffino S. Dyar K. A. Ciciliot S. Blaauw B. Sandri M. (2013). Mechanisms regulating skeletal muscle growth and atrophy. Febs J. 280 (17), 4294–4314. 10.1111/febs.12253 23517348

[B85] Serrano A. L. Muñoz-Cánoves P. (2010). Regulation and dysregulation of fibrosis in skeletal muscle. Exp. Cell Res. 316 (18), 3050–3058. 10.1016/j.yexcr.2010.05.035 20570674

[B86] Shirasawa H. Matsumura N. Shimoda M. Oki S. Yoda M. Tohmonda T. (2017). Inhibition of PDGFR signaling prevents muscular fatty infiltration after rotator cuff tear in mice. Sci. Rep. 7, 41552. 10.1038/srep41552 28139720 PMC5282512

[B87] Shirasawa H. Matsumura N. Yoda M. Okubo K. Shimoda M. Uezumi A. (2021). Retinoic acid receptor agonists suppress muscle fatty infiltration in mice. Am. J. Sports Med. 49 (2), 332–339. 10.1177/0363546520984122 33428447

[B88] Stamiris D. Valasidis A. Cheva A. Papavasiliou K. Stamiris S. Potoupnis M. (2024). Interventions used to mitigate muscle fatty degeneration following the repair of massive rotator cuff tears. A systematic review of animal studies. Orthop. Traumatol. Surg. Res. 110 (1), 103723. 10.1016/j.otsr.2023.103723 37879533

[B89] Thomazeau H. Boukobza E. Morcet N. Chaperon J. Langlais F. (1997). Prediction of rotator cuff repair results by magnetic resonance imaging. Clin. Orthop. Relat. Res. 344, 275–283. 10.1097/00003086-199711000-00027 9372778

[B90] Tidball J. G. (2017). Regulation of muscle growth and regeneration by the immune system. Nat. Rev. Immunol. 17 (3), 165–178. 10.1038/nri.2016.150 28163303 PMC5452982

[B91] Tintignac L. A. Brenner H. R. Rüegg M. A. (2015). Mechanisms regulating neuromuscular junction development and function and causes of muscle wasting. Physiol. Rev. 95 (3), 809–852. 10.1152/physrev.00033.2014 26109340

[B92] Uezumi A. Fukada S. Yamamoto N. Takeda S. Tsuchida K. (2010). Mesenchymal progenitors distinct from satellite cells contribute to ectopic fat cell formation in skeletal muscle. Nat. Cell Biol. 12 (2), 143–152. 10.1038/ncb2014 20081842

[B93] Uezumi A. Ito T. Morikawa D. Shimizu N. Yoneda T. Segawa M. (2011). Fibrosis and adipogenesis originate from a common mesenchymal progenitor in skeletal muscle. J. Cell Sci. 124 (21), 3654–3664. 10.1242/jcs.086629 22045730

[B94] Valencia A. P. Lai J. K. Iyer S. R. Mistretta K. L. Spangenburg E. E. Davis D. L. (2018). Fatty infiltration is a prognostic marker of muscle function after rotator cuff tear. Am. J. Sports Med. 46 (9), 2161–2169. 10.1177/0363546518769267 29750541 PMC6397750

[B95] Wang Z. Liu X. Davies M. R. Horne D. Kim H. Feeley B. T. (2018a). A mouse model of delayed rotator cuff repair results in persistent muscle atrophy and fatty infiltration. Am. J. Sports Med. 46 (12), 2981–2989. 10.1177/0363546518793403 30198747 PMC6730552

[B96] Wang Z. Feeley B. T. Kim H. T. Liu X. (2018b). Reversal of fatty infiltration after suprascapular nerve compression release is dependent on UCP1 expression in mice. Clin. Orthop. Relat. Res. 476 (8), 1665–1679. 10.1097/CORR.0000000000000335 30020151 PMC6259770

[B97] Wang Z. Liu X. Jiang K. Kim H. Kajimura S. Feeley B. T. (2020). Intramuscular brown fat activation decreases muscle atrophy and fatty infiltration and improves gait after delayed rotator cuff repair in mice. Am. J. Sports Med. 48 (7), 1590–1600. 10.1177/0363546520910421 32282238

[B98] Wang Z. Liu X. Liu M. Jiang K. Kajimura S. Kim H. (2021). β(3)-Adrenergic receptor agonist treats rotator cuff fatty infiltration by activating beige fat in mice. J. Shoulder Elb. Surg. 30 (2), 373–386. 10.1016/j.jse.2020.06.006 32599287 PMC7765745

[B99] Wosczyna M. N. Konishi C. T. Perez Carbajal E. E. Wang T. T. Walsh R. A. Gan Q. (2019). Mesenchymal stromal cells are required for regeneration and homeostatic maintenance of skeletal muscle. Cell Rep. 27 (7), 2029–2035.e2025. 10.1016/j.celrep.2019.04.074 31091443 PMC7034941

[B100] Wynn T. A. Vannella K. M. (2016). Macrophages in tissue repair, regeneration, and fibrosis. Immunity 44 (3), 450–462. 10.1016/j.immuni.2016.02.015 26982353 PMC4794754

[B101] Yamaguchi K. Tetro A. M. Blam O. Evanoff B. A. Teefey S. A. Middleton W. D. (2001). Natural history of asymptomatic rotator cuff tears: a longitudinal analysis of asymptomatic tears detected sonographically. J. Shoulder Elb. Surg. 10 (3), 199–203. 10.1067/mse.2001.113086 11408898

[B102] Yoon J. P. Park S. J. Kim D. H. Lee H. J. Kim J. Y. Pham D. T. (2026). GLP-1 receptor agonist suppresses fatty infiltration while improving range of motion and electromyographic function in a chronic rotator cuff tear rat model. J. Shoulder Elb. Surg. 35 (7), 1800–1808. 10.1016/j.jse.2025.12.019 41644032

[B103] Zanetti M. Gerber C. Hodler J. (1998). Quantitative assessment of the muscles of the rotator cuff with magnetic resonance imaging. Invest. Radiol. 33 (3), 163–170. 10.1097/00004424-199803000-00006 9525755

[B104] Zbinden J. Michalewska K. Ho S. W. Nabergoj M. Ibrahim M. Chiu J. C. (2026). Muscle edema of the rotator cuff: a systematic review of characteristics and associated pathologies from the LaTour group. EFORT Open Rev. 11 (4), 338–348. 10.1530/eor-2025-0096 41945591 PMC13087873

[B105] Zhang Y. Li X. Zhang R. Wang M. Wang T. Liu G. (2025). Tanshinone IIA suppresses fibro-adipogenic progenitor differentiation and attenuates fat infiltration in rotator cuff injury via Wnt/β-catenin pathway. Regen. Ther. 30, 584–594. 10.1016/j.reth.2025.08.004 40837862 PMC12362028

[B106] Zhou H. Chen C. Hu H. Jiang B. Yin Y. Zhang K. (2023). High-intensity interval training improves fatty infiltration in the rotator cuff through the β3 adrenergic receptor in mice. Bone Jt. Res. 12 (8), 455–466. 10.1302/2046-3758.128.bjr-2022-0309.r2 37524338 PMC10390263

[B107] Zhou H. Lin X. Feng S. Zhu S. Zhou H. Chen H. (2024). Metformin mitigates adipogenesis of fibro-adipogenic progenitors after rotator cuff tears via activating mTOR/ULK1-mediated autophagy. Am. J. Physiol. Cell Physiol. 326 (6), C1590–c1603. 10.1152/ajpcell.00034.2024 38586878 PMC11371331

