# Supplementary material for: Tendon-muscle-nerve disconnection in chronic large and massive posterosuperior rotator cuff tears: a targeted narrative mechanistic review and hypothesis-generating framework
Source: Front Cell Dev Biol. 2026 Sep 14;14:1928020. doi: 10.3389/fcell.2026.1928020 (PMC13617102; doi:10.3389/fcell.2026.1928020)
Supplement: Supplementary file 1 [file Table1.docx]

Supplementary Material

Tendon-Muscle-Nerve Disconnection in Chronic Massive Posterosuperior Rotator Cuff Tears: A Mechanistic Review and Research Framework

Supplementary Table S1. Search strategy and eligibility framework for the narrative review.

| **Component** | **Specification** |
| --- | --- |
| Design | Targeted narrative mechanistic review intended for conceptual synthesis and hypothesis generation, not pooled effect estimation. |
| Databases, dates and language | PubMed, Scopus and Web of Science; database inception to May 30, 2026; English-language records. |
| PubMed | (("Rotator Cuff Injuries"[Mesh] OR rotator cuff tear*[tiab] OR massive rotator cuff tear*[tiab] OR posterosuperior rotator cuff[tiab]) AND (fatty infiltration[tiab] OR muscle atrophy[tiab] OR fibrosis[tiab] OR suprascapular neuropath*[tiab] OR electromyograph*[tiab] OR neuromuscular junction[tiab] OR fibro-adipogenic progenitor*[tiab] OR extracellular matrix[tiab] OR macrophage*[tiab] OR exosome*[tiab] OR extracellular vesicle*[tiab] OR muscle edema[tiab] OR force transmission[tiab] OR beige adipogenesis[tiab] OR GLP-1 receptor agonist*[tiab])). |
| Scopus | TITLE-ABS-KEY(("rotator cuff tear*" OR "massive rotator cuff tear*" OR "posterosuperior rotator cuff") AND ("fatty infiltration" OR "muscle atrophy" OR fibrosis OR "suprascapular neuropath*" OR electromyograph* OR "neuromuscular junction" OR "fibro-adipogenic progenitor*" OR "extracellular matrix" OR macrophage* OR exosome* OR "extracellular vesicle*" OR "muscle edema" OR "force transmission" OR "beige adipogenesis" OR "GLP-1 receptor agonist*")). |
| Web of Science | TS=(("rotator cuff tear*" OR "massive rotator cuff tear*" OR "posterosuperior rotator cuff") AND ("fatty infiltration" OR "muscle atrophy" OR fibrosis OR "suprascapular neuropath*" OR electromyograph* OR "neuromuscular junction" OR "fibro-adipogenic progenitor*" OR "extracellular matrix" OR macrophage* OR exosome* OR "extracellular vesicle*" OR "muscle edema" OR "force transmission" OR "beige adipogenesis" OR "GLP-1 receptor agonist*")). |
| Eligibility | Included human imaging/outcome, electrodiagnostic and histological studies; relevant rotator cuff animal models; and foundational skeletal-muscle biology directly informing a prespecified framework component. Excluded isolated technical reports, non-mechanistic case reports and non-cuff studies without a direct interpretive role. |
| Selection and citation tracking | Relevance-based title/abstract review followed by full-text assessment; backward and forward citation tracking from key studies and recent reviews. No duplicate independent screening was performed. |
| Synthesis | Narrative integration by mechanism, biological stage and evidence tier. Conflicting evidence and translational limitations were retained explicitly; no pooled effect estimate or formal risk-of-bias score was calculated. |

Supplementary Table S2. SANRA-informed self-audit of the revised narrative review.

| **SANRA domain** | **How addressed** | **Location** |
| --- | --- | --- |
| Importance to readership | Clinical problem and knowledge gap are stated; the framework targets unexplained weakness despite tendon healing. | Abstract and Introduction |
| Concrete aims | The review aims to separate mechanism from stage, calibrate evidence and generate falsifiable hypotheses. | Abstract, Introduction and Box 1 |
| Literature search | Databases, dates, language, exact search strings, eligibility and non-duplicate selection are disclosed. | Review approach and Table S1 |
| Referencing | All mechanistic claims are linked to the relevant evidence layer; references remain sequential. | Main text and References |
| Scientific reasoning | Direct human, associative, animal causal, extrapolative and hypothesis tiers are distinguished; conflicting evidence is retained. | Table 1 and Limitations |
| Presentation of data | Mechanism and stage are separated; novelty, translational readiness and validation endpoints are tabulated. | Tables 1-3 and Figures 1-3 |
| Framework source | SANRA framework.[1] | Reference [1] |

Supplementary Table S3. Proposed experimental matrix for validating the two-dimensional TMN framework.

| **Group** | **Purpose** | **Key endpoints** |
| --- | --- | --- |
| Sham control | Define baseline muscle, nerve and stromal states. | Regional fat fraction, edema-like T2 signal, force, NMJ morphology, FAP and macrophage-state profiles. |
| Isolated tendon injury | Test mechanical unloading without direct nerve injury. | Peripennate/myotendinous fat distribution, load-response markers, FAP/ECM state. |
| Isolated nerve injury | Test a defined peripheral neural lesion without tendon unloading. | EMG/NCS, motor-unit and NMJ changes, regional fat, UCP1 and FAP state. |
| Combined injury | Test interaction between mechanical and peripheral neural injury. | Severity, fibrosis, force, regional mapping and spatial transcriptomics. |
| Delayed repair | Test stage-dependent reversibility after mechanical reconnection. | Force recovery, fat/fibrosis trajectory, repair integrity and stromal state. |
| Nerve release/decompression | Test neural reversibility in a documented compression model. | Electrophysiological recovery, fat trajectory and NMJ/UCP1 markers. |
| Repair plus biologic modulation | Test combined restoration and fate redirection in an intermediate-stage model. | FAP/ECM and immune state, fat fraction, strength, endurance, tendon healing and safety. |

**Reference**

1.	Baethge C, Goldbeck-Wood S, Mertens S: **SANRA-a scale for the quality assessment of narrative review articles**. *Res Integr Peer Rev* 2019, **4**:5.
